# Transcriptome Analysis of the Growth-Promoting Effect of Large Macrofungal Sclerotium Powder on *Lentinula edodes* and *Pleurotus eryngii* Strains

**DOI:** 10.3390/jof10120808

**Published:** 2024-11-21

**Authors:** Zhanghu Chen, Ruiheng Yang, Yan Li, Lihua Tang, Huiyang Xiong, Dapeng Bao, Ting Guo

**Affiliations:** 1 National Research Center for Edible Fungi Biotechnology and Engineering, Key Laboratory of Applied Mycological Resources and Utilization, Ministry of Agriculture, Shanghai Key Laboratory of Agricultural Genetics and Breeding, Institute of Edible Fungi, Shanghai Academy of Agricultural Sciences, Shanghai 201403, China; chenzhanghu1998@163.com (Z.C.); yangrh@saas.sh.cn (R.Y.); liyan03@saas.sh.cn (Y.L.); lhtang2007@163.com (L.T.); xionghuiyang@saas.sh.cn (H.X.); 2 College of Food Science, Shanghai Ocean University, Shanghai 201306, China; 3 Yunnan Key Laboratory of Gastrodia and Fungi Symbiotic Biology, Zhaotong University, Zhaotong 657000, China

**Keywords:** *Lentinula edodes*, *Pleurotus eryngii*, sclerotium, transcriptome, medium composition, energy metabolism

## Abstract

In the industrial production of *Lentinula edodes* and *Pleurotus eryngii*, slow growth of the mother seed and insufficient hyphal vitality can significantly affect the cultivation process. To shorten the growth period on traditional PDA medium, two strains of *L. edodes* and *P. eryngii* were cultured with different proportions of *P*. *tuber-regium* and *Wolfiporia hoelen* sclerotium powders added into the medium to investigate the effect on the mycelial growth. Compared to the PDA, the addition of sclerotia powder significantly enhanced the growth of mycelia, with an optimal addition ratio of 2%. Transcriptome sequencing was performed after culturing *L. edodes* and *P. eryngii* on PDA, PDA with 2% *P. tuber-regium* sclerotium powder, and PDA with 2% *W. hoelen* sclerotium powder. GO enrichment analysis of the differentially expressed genes (DEGs) of *L. edodes* and *P. eryngii* strains cultured in the sclerotia powder media showed significant changes in oxidoreductase and glucosidase activities. Changes were observed in KEGG annotation for carbohydrate metabolism, glycolysis, pyruvate metabolism, and other energy metabolic pathways. Moreover, carbohydrate-active enzyme (CAZyme) family genes were predominantly upregulated. The increase in the activity of CAZyme and oxidoreductases promotes the degradation of nutrients in the sclerotia into small-molecule substances, which explains why the sclerotia powder culture medium promotes mycelial growth.

## 1. Introduction

Cultivation of edible mushroom strains on solid plate media is a fundamental experiment conducted in laboratories and factories for basic research and large-scale cultivation. The most commonly used media for fungi are potato dextrose agar (PDA), yeast extract agar, and malt extract agar [1]. However, these media were initially designed for the cultivation of filamentous fungi and may not be suitable for the growth of most edible mushroom strains. Consequently, when using these media to cultivate edible mushrooms, issues such as slow mycelial growth, pigment production, mycelial aging, and sparsely branched mycelia may occur, which can greatly limit mycelial proliferation and adversely affect the maintenance of strain characteristics [2,3].

Most cultivated edible mushroom species in the wild utilise substrates rich in lignocellulose such as decaying wood, dead branches and leaves, and straw. During the long evolutionary process, the mycelium has developed an adaptive mechanism for digestion and absorption, secreting large amounts of carbohydrate-degrading enzymes to decompose these substrates [4]. Therefore, during the process of cultivating edible mushroom strains in the laboratory or factory, the addition, reduction, or replacement of substrates closely related to carbohydrate enzyme metabolism may affect mycelial growth to varying degrees. Previous studies have mainly focused on two types of substrates for the selection and improvement of medium formulations. The first is a single, well-defined carbon source. Yuan et al. [5] found that maltose was the optimal carbon source for the cultivation of wild *Lentinula edodes* strains using orthogonal experiments. Yan et al. [6] identified glucose as the fastest-growing carbon source for *Pleurotus eryngii* mycelia. The second strategy consists of natural substrates mainly constituting carbohydrates, such as crops, crop residues, or waste materials, which are used as alternatives or complements to PDA. A medium consisting of a wheat straw and wheat grain mixture promoted the growth of *P*. *ostreatus* and increased the protein content and protease activity of the mycelium [7]. Materials such as rice straw [8], dried manure [9], corn flour, and compost [10] promoted the growth and development of straw-rot mushrooms such as *Agaricus bisporus* and *Stropharia rugosoannulata*, and improved strain degeneration. Cotton seed hulls, mulberry sawdust, and bran [11,12] promote the growth and development of most wood-decaying fungi, such as *L. edodes* and *P. eryngii*. The supplements of an appropriate amount of sawdust to the PDA medium promoted the growth of *L*. *edodes* [13]. In addition, adding an appropriate proportion of rice husks [14], peanut shells [15,16], honeysuckle vines [17], bran [18,19,20], and other similar wastes to the medium can promote the growth of edible mushrooms to varying degrees.

The aforementioned commonly used natural substrates from plants primarily comprise polysaccharides. Polysaccharides are abundant not only in plants but also in fungi, such as the fungal sclerotium. However, the research on the use of fungal polysaccharides as a source of nutrients for edible mushrooms is limited. The sclerotium of macro fungi is a hard mycelial structure formed by interwoven mycelia [21]. The morphology is variable, nutrient-rich, and can maintain a dormant state in unfavourable environments, such as drought and lack of hosts [22]. Among the higher fungi of the Basidiomycota and Ascomycota phyla, several fungi are capable of forming sclerotia, and some can form large sclerotia, such as *Omphalia lapidescens*, *P*. *tuber-regium* (hereinafter referred to as PTR) [23], *Polyporus umbellatus*, and *Wolfiporia hoelen* (hereinafter referred to as WH) [24]. Compared to other sclerotia, PTR and WH have large sclerotia with high biomass and their ingredients are now more clearly defined [25]. Furthermore, they are characterised by a high concentration of polysaccharides. The primary component of WH is β-poria polysaccharide, which accounts for 60–90% of the dry weight of the sclerotia [26,27], and the primary component of PTR is β-glucan, accounting for 61.7–80% of the sclerotium [28,29,30]. Compared to natural plant substrates, such as wood chips and straw, sclerotia contain high levels of carbohydrates, which can provide abundant carbon sources for mycelial growth. Chen et al. [31] found that the addition of a small amount of sclerotium powder can promote the mycelial growth of a variety of edible mushrooms, especially for some slow-growing edible mushrooms such as *Lyophyllum decastes* and *Phlebopus portentosus*.

When using PDA plates for subculturing *L. edodes* and *P. eryngii*, challenges such as reduced mycelial growth rate, sparse mycelia, and weakened growth become prominent. These factors directly affect the subsequent cultivation process and greatly hinder the healthy development of the edible mushroom industry. In this study, the *L. edodes* and *P. eryngii* strains were selected as the experimental materials. The aim of this study was to compare the growth rate and status of mycelia on PDA medium supplemented with PTR and WH sclerotia powder, and to explore whether edible mushrooms can utilise nutrients from sclerotia. Additionally, differentially expressed genes (DEGs) during mycelial growth were further analysed by transcriptome sequencing to elucidate the biological mechanisms by which edible mushrooms utilise nutrients in the sclerotia.

## 2. Materials and Methods

### 2.1. Fungal Strains and Cultivation

The strains used in this experiment, *L. edodes* (strain No. Wuxiang w1) and *P. eryngii* (strain No. Xinghan), were obtained from the Edible Fungi Research Institute of Shanghai Academy of Agricultural Sciences. The PTR sclerotium was purchased from Jiangxi Licai Edible Fungi Co., Ltd. (Fuzhou, China), and WH sclerotium was purchased from Anhui Agricultural Development Co., Ltd. (Hefei, China). The strains were grown in PDA medium, PDA medium with 0.5%, 1%, 2%, 3%, and 4% PTR, or WH sclerotia powders, respectively. The mycelium in the treatment experiments was incubated at 25 °C in total darkness for 7 days and then harvested for RNA extraction and transcriptome sequencing. Each treatment experiment was carried out in independent biological triplicates under the same experimental conditions.

### 2.2. Determination of Mycelial Growth Rate and DAPI Nuclear Staining

During the mycelial growth process, the growth rate of mycelia was measured using the cross method [32]. A sterile cover glass was inserted at a 45° angle at the edge of the colony, and the dishes were incubated at 25 °C in an incubator until the mycelium grew to 2/3 of the dish (consistent with the time of measuring the growth rate). Cover glasses with well-developed mycelia were prepared to observe the mycelial morphology. Then, 20 μL of DAPI staining solution was added to the centre of each cover glass. The cover glasses were covered with a staining solution and left for 10–15 min in the dark. Excess staining solution was removed using an absorbent paper. The density of the mycelial nuclei was observed under a fluorescent inverted microscope (Zeiss LSM880, Carl Zeiss AG, Oberkochen, Germany) at 40× magnification, and the thickness of the mycelial tip was measured. The experimental data were analysed for statistical significance (*p* < 0.05).

### 2.3. RNA Extraction, Library Construction, and RNA Sequencing

For transcriptome sequencing, samples of *L. edodes* and *P. eryngii* were cultured on medium with the optimal concentration of *P. tuber-regium* and *W. hoelen* sclerotium powder, respectively. In order to determine the optimal concentration to be added in the medium, different media were prepared and the selectivity of medium was determined by comparing the growth rate. Samples collected were frozen in liquid nitrogen immediately and stored at −80 °C before RNA extraction. Total RNA was extracted using the FastPure Universal Plant Total RNA Isolation Kit (Lot #RC411; Vazyme, Nanjing, China) following the manufacturer’s instructions, and a Nanodrop 2000 spectrophotometer was used to evaluate the quantity and quality of the extracted RNA. RNA integrity was checked using agarose gel electrophoresis. After the samples passed the qualification test, library construction and transcriptome sequencing were performed using 150 bp paired-end read libraries on the DNBSEQ-T7 platform (MGI) by Personal Biotechnology Co., Ltd., Shanghai, China.

### 2.4. Transcriptome Data Analysis

Quality assessment of sequencing data was performed. After the raw data were obtained, Trimmomatic [33] was used to filter adapter and low-quality sequences, remove reads with low-quality bases (Q < 30), and trim sequences shorter than 35 nt after quality trimming to obtain high-quality clean data. HISAT2 2.2.1 [34] software was used to align the filtered reads to the reference genome for sequence alignment and assembly. The Q30 and mapping rates of the clean data for *L. edodes* and *P. eryngii* to the reference genome were used to evaluate the quality of transcriptome sequencing.

Analysis of gene expression and identification of differentially expressed genes (DEGs) was conducted using StringTie v2.2.3 [35] software to quantify gene expression levels in the samples, count the number of sequencing sequences (reads) located in the genome or exon regions, and the transcript per million (TPM) value was calculated for normalisation of gene length and sequencing depth. DESeq 3.20 software [36] was used for data processing to identify significantly DEGs. The screening criteria were set as q Value < 0.05 and fold change |Fold Change| > 2.

Functional annotation analysis was performed using the Gene Ontology (GO) database (http://www.geneontology.org, accessed on 12 August 2023) and Kyoto Encyclopedia of Genes and Genomes (KEGG) database (http://www.kegg.jp, accessed on 12 August 2023) to annotate the DEGs. R scripts topGO and clusterProfiler were used for functional enrichment analysis of DEGs, and *p*-values (Q value) < 0.05 were considered to be significant.

For annotation and analysis of DEGs in carbohydrate-active enzyme (CAZyme) families [37], the genome sequences of DEGs were downloaded from NCBI (http://ncbi.nlm.nih.gov/, accessed on 12 August 2023) and dbCAN3 (https://bcb.unl.edu/dbCAN2/, accessed on 12 August 2023) was used to search for CAZyme domains. The parameters were set as E value < 1 × 10^−15^ and coverage > 0.35, and the results were summarised and analysed.

### 2.5. Selection of Key DEGs and Analysis by Quantitative Real-Time PCR

Common DEGs between *L. edodes* and *P. eryngii* were selected for quantitative real-time PCR (qRT-PCR) analysis to validate the reliability of the transcriptome data. The primers were designed using the Primer Premier 6 software (Premier Biosoft International, Palo Alto, CA, USA), and the actin genes of *A. bisporus* [38] and *P. eryngii* [39] were selected as internal reference genes. Information of the primers used is presented in Table S1. For reverse transcription of RNA, utilize the HiScript III RT Super Mix for qPCR Kit as per the manufacturer’s protocol (Vazyme #R323, Nanjing, China). After verifying the specificity of the qRT-PCR primers using cDNA as a template, quantitative fluorescence detection was performed. Three replicates were performed for each gene, and the relative gene expression levels were calculated using the 2^−ΔΔCt^ method [40].

### 2.6. Statistical Analysis

The obtained experimental data were analysed for significance using SPSS software (IBM SPSS Statistics for Windows, Version 25.0, IBM Corp, Armonk, NY, USA), and GraphPad Prism version 8.0 (GraphPad Software, Inc., San Diego, CA, USA) was used to plot the graphs. Each value in the graphs represents the mean ± standard deviation, and different letters in the graphs indicate significant differences between treatments (*p* < 0.05).

## 3. Results

### 3.1. Mycelial Growth Rate with Different Added Ratios of Sclerotium Powder

**Figure 1:**
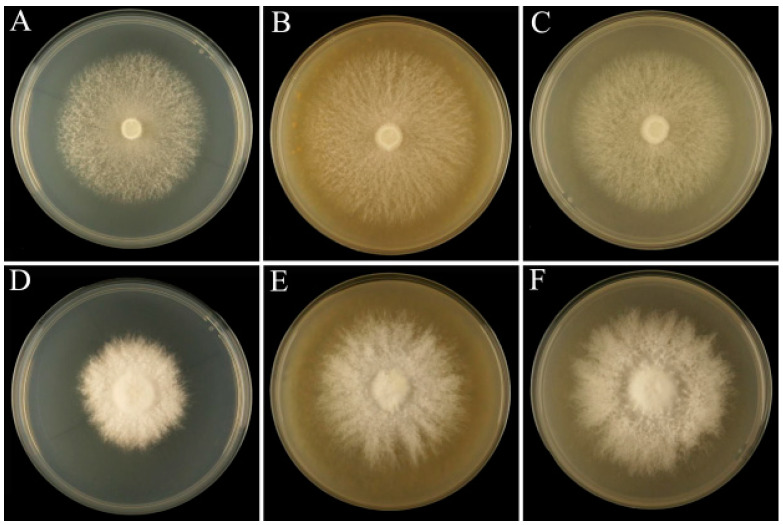
Comparison of mycelial growth of *Lentinula edodes* and *Pleurotus eryngii* in potato dextrose agar (PDA) medium, PDA with 2% *P. tuber-regium* sclerotium powder, and PDA with 2% *Wolfiporia hoelen* sclerotium powder. (**A**–**C**): *Lentinula edodes*. (**D**–**F**): *Pleurotus eryngii*.

**Figure 2:**
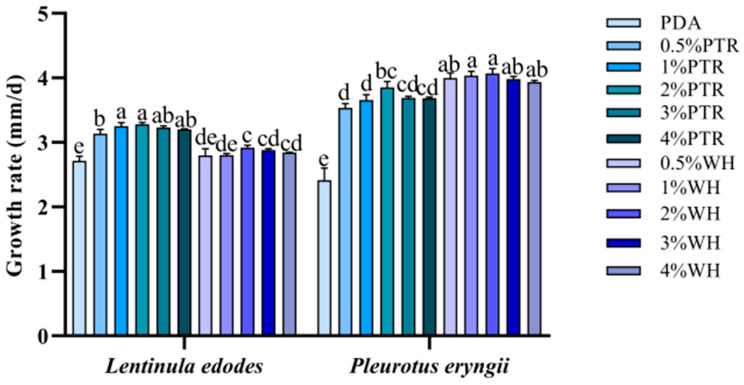
Growth rates of *Lentinula edodes* and *Pleurotus eryngii* strains on different nutrient media. Different lowercase letters represent significant differences between samples (*p* < 0.05). PTR: mycelia growth in PDA with *P. tuber-regium* sclerotium powder. WH: mycelia growth in PDA with *Wolfiporia hoelen* sclerotium powder.

Compared with the control PDA medium, the mycelia in the plates supplemented with sclerotium powder appeared white and robust, and colony morphology was improved (Figure 1). The *L. edodes* strain showed a similar growth rate when supplemented with 0.5–4% sclerotium powder. The most significant increase in growth was observed in 2% PTR medium and 2% WH medium, with the maximum speed of mycelial growth increasing from 2.72 mm/d in the PDA medium to 3.28 mm/d and 2.92 mm/d, respectively (Figure 2). For *P. eryngii*, the growth rate of the colony was higher in media supplemented with 0.5–4% PTR and 0.5–4% WH sclerotium powders than that in the PDA medium. The most significant increase in growth was observed in 2% PTR medium and 2% WH medium, with the maximum mycelial growth rate increasing from 2.41 mm/d in the PDA medium to 3.85 mm/d and 4.06 mm/d, respectively.

### 3.2. Microscopic Observation of Mycelial Morphology

**Figure 3:**
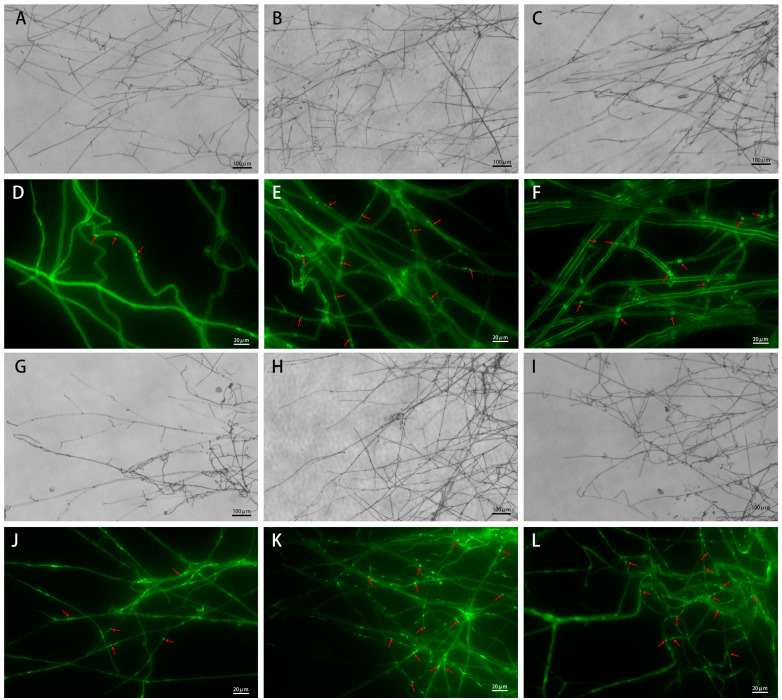
Microscopic morphology of the mycelium of *Lentinula edodes* (Le) and *Pleurotus eryngii* (Pe) on different media. (**A**): Le-PDA. (**B**): 2% Le-PTR. (**C**): 2% Le-WH. (**D**): Le-PDA. (**E**): 2% Le-PTR. (**F**): 2% Le-WH. (**G**): Pe-PDA. (**H**): 2% Pe-PTR. (**I**): 2% Pe-WH. (**J**): Pe-PDA. (**K**): Pe-PTR. (**L**): Pe-WH. The red arrows indicate nuclei.

Compared with PDA, the mycelia of *L. edodes* and *P. eryngii* grown on PDA supplemented with sclerotium powder appeared dense, with increased branching at the tips of the mycelium. After DAPI staining, the number of nuclei in the mycelia grown on sclerotium powder-supplemented media (with the addition of 2% sclerotium powder) was significantly higher than that on the PDA medium (Figure 3).

### 3.3. Analysis of DEGs

The Q30 bases of *L. edodes* and *P. eryngii* were all above 85% and 90%, respectively, indicating good sequencing quality. The mapping rates of *L. edodes* and *P. eryngii* to the reference genome ranged from 71.24% to 85.95% and from 86.74% to 88.46%, respectively, indicating that most of the DEGs could be identified and used for subsequent analysis.

**Figure 4:**
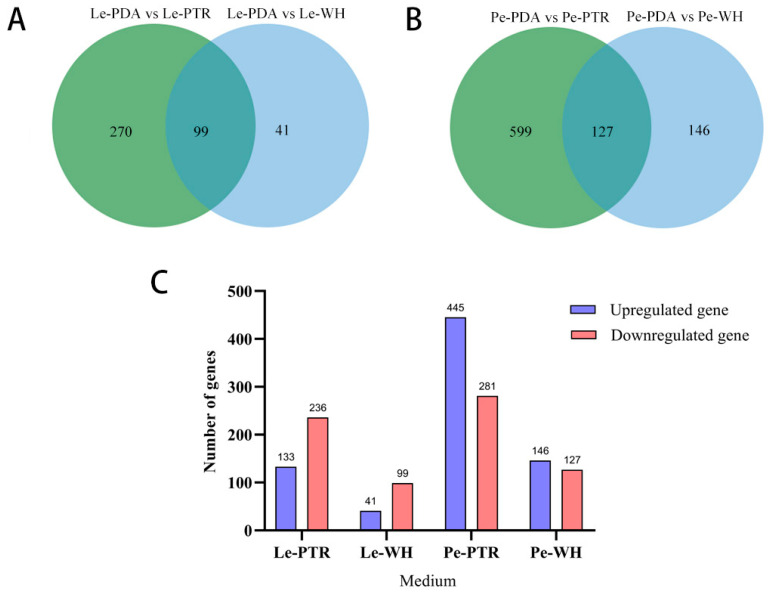
Venn analyses based on the total gene expression profiles. (**A**,**B**): Venn diagram exhibiting the DEGs’ distribution in different libraries. (**C**): Number of DEGs in *L. edodes* and *P. eryngii*.

Based on the screening criteria, in the *L. edodes* transcriptome data, 410 DEGs were identified, of which 369 DEGs were present in the Le-PDA vs. Le-PTR comparison, with 133 upregulated and 263 downregulated genes. The number of DEGs in the Le-PDA vs. Le-WH group was 140, with 41 upregulated and 99 downregulated genes (Figure 4). A total of 872 DEGs were identified in the Pe transcriptome data. There were 726 DEGs in the Pe-PDA vs. Pe-PTR comparison, with 455 upregulated and 281 downregulated genes. In Pe-PDA vs. Pe-WH, there were 273 DEGs, with 146 upregulated and 127 downregulated genes. Using BioEdit, the protein sequences of the 99 common DEGs in *L. edodes* were compared to those of the 127 common DEGs in *P. eryngii* (Table S1). Thirteen protein sequences of *L. edodes* had high similarity with twenty-four protein sequences of *P. eryngii*, mainly involving cytochrome P450 and NAD (P)-binding proteins.

### 3.4. Functional Annotation of DEGs

**Figure 5:**
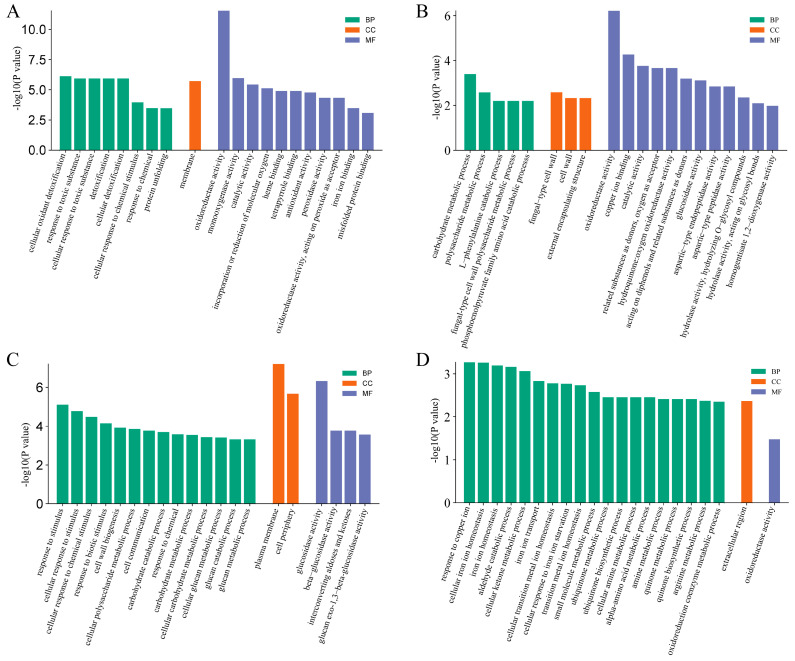
GO annotation of co-induced DEGs. (**A**): Le-PDA vs. Le-PTR. (**B**): Le-PDA vs. Pe-WH. (**C**): Pe-PDA vs. Pe-PTR. (**D**): Pe-PDA vs. Pe-WH. BP: biological process. CC: cellular component. MF: molecular function.

GO analysis was performed on the mycelia cultured on media with PTR and WH sclerotium powders. GO functional enrichment analysis was conducted on screened DEGs to describe their biological functions from three perspectives: biological process (BP), cellular component (CC), and molecular function (MF). In the PTR medium, *L. edodes* and *P. eryngii* strains were enriched in oxidoreductase activity and response to chemicals. In the WH medium, *L. edodes* and *P. eryngii* were enriched in oxidoreductase activity (Figure 5).

**Figure 6:**
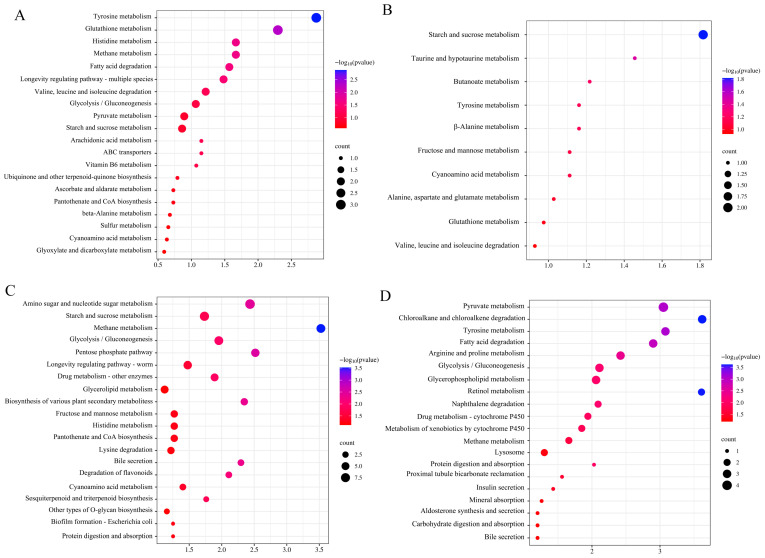
KEGG annotation of co-induced differentially expressed genes. (**A**): Le-PDA vs. Le-PTR. (**B**): Le-PDA vs. Le-WH. (**C**): Pe-PDA vs. Pe-PTR. (**D**): Pe-PDA vs. Pe-WH.

Common DEGs were annotated using KEGG metabolic pathways. *Lentinula edodes* and *P. eryngii* were enriched in starch and sucrose metabolism, pantothenate and CoA biosynthesis, and glycolysis/gluconeogenesis pathways in the PTR medium. In the WH medium, they were enriched in tyrosine metabolism and energy-related metabolic pathways (Figure 6).

### 3.5. Expression Analysis of CAZYme Family Genes in DEGs

**Figure 7:**
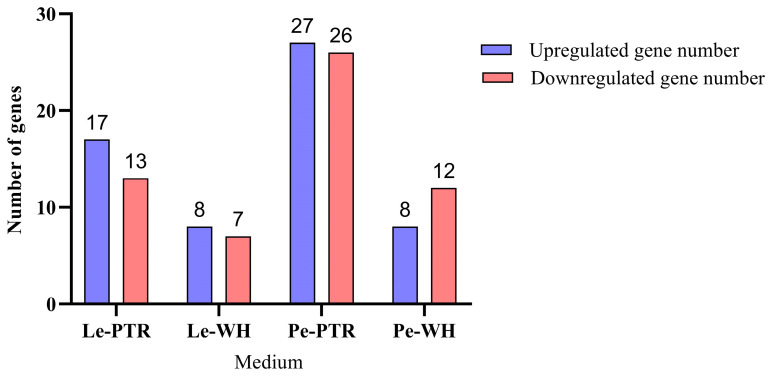
Differential gene expression in the CAZYme family.

Based on GO and KEGG annotations, the DEGs were mainly enriched in carbohydrate metabolism and oxidative reduction reactions. Therefore, CAZYme family genes were analysed. In the PTR medium, *L. edodes* mycelia had 30 CAZYme family genes in the DEGs, with 17 upregulated and 13 downregulated genes. In the case of mycelium in the WH medium, 15 CAZYme family genes were present among the DEGs, with 8 upregulated and 7 downregulated genes (Figure 7). *Pleurotus eryngii* mycelia cultured in PTR medium had 53 CAZYme family genes in the DEGs, with 27 upregulated and 26 downregulated genes. The mycelium cultured in the WH medium had 20 CAZYme family genes among the DEGs, with 8 upregulated and 12 downregulated genes.

**Figure 8:**
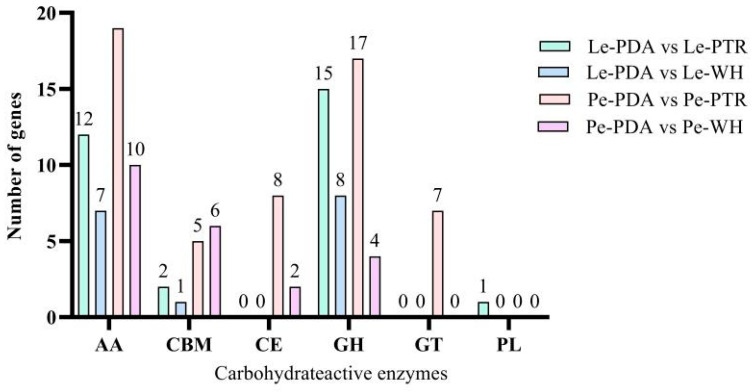
Number of significant DEGs in the CAZYme family.

Among the 99 DEGs induced by both types of sclerotia powder in *L. edodes*, 8 were CAZYme family genes. Among these, five genes belonged to the auxiliary activity (AA) family and three genes to the glycoside hydrolases (GH) family. Four oxidoreductase genes were upregulated (GAW03759, GAW00335, GAW06442, and GAW08622), three oxidoreductase genes were downregulated (GAV99068, GAW05002, and GAW05592), and one cellulase gene was downregulated (GAW07534). A total of 8 CAZYme family genes were present among the 127 DEGs induced by both types of sclerotia powder in *P. eryngii*. Among these, five genes belonged to the AA family and three to the carbohydrate-binding modules (CBM) family. Four oxidoreductase genes were upregulated (BDN71DRAFT_1055494, BDN71DRAFT_1607096, BDN71DRAFT_1435398, and BDN71DRAFT_1445894), three oxidoreductase genes were downregulated (BDN71DRAFT_1483600, BDN71DRAFT_416606, and BDN71DRAFT_1447255), and one hemicellulase gene was downregulated (BDN71DRAFT_106407) (Figure 8).

### 3.6. Validation of Results by Real-Time Fluorescence Quantitative PCR

Eight CAZYme genes that were differentially expressed in both *L. edodes* and *P. eryngii* were selected for qRT-PCR analysis. Tables S2 and S3 show the DEGs and corresponding primer information for *L. edodes* and *P. eryngii*, respectively.

**Figure 9:**
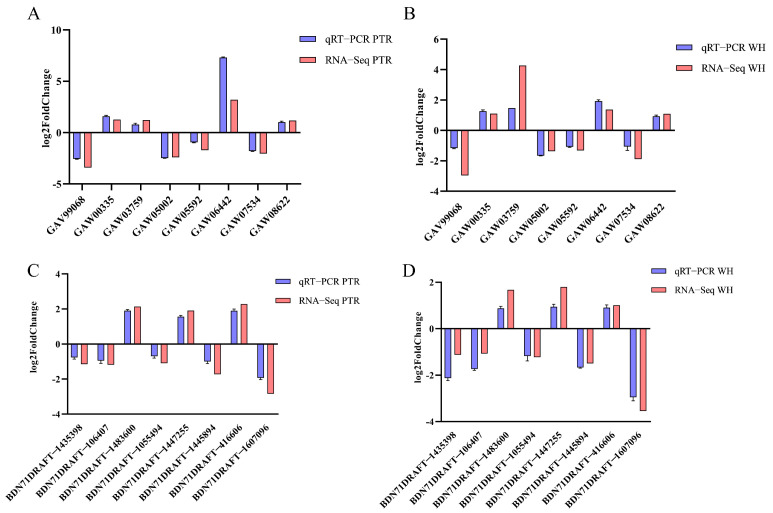
qRT-PCR validation results of DEGs. (**A**): Pe-PDA vs. Pe-WH. (**B**): Pe-PDA vs. Pe-WH. (**C**): Le-PDA vs. Le-WH. (**D**): Pe-PDA vs. Pe-WH.

The eight DEGs identified by the qRT-PCR results were consistent with those from the transcriptome analysis results (Figure 9), indicating the reliability of the transcriptome analysis data in this study.

## 4. Discussion

The results showed that adding a certain proportion of PTR or WH sclerotium powder in PDA medium (the optimal proportion was 2%) could promote the mycelial growth of *L. edodes* and *P. eryngii*, enhance the mycelial growth rate, and significantly shorten the culture time on the plate. Studies have shown that long-term use of PDA medium to subculture edible fungi can inhibit the physiological metabolic pathway of mycelia to decompose the substrate, resulting in the degradation of strains [41]. The growth and development of edible fungal mycelia are promoted by adding appropriate amounts of polysaccharides to the PDA medium. For example, a low concentration of *Astragalus* water extract promoted mycelial growth in five edible fungi, including *P. eryngii* and *L. edodes* [42]; the main component of *Astragalus* water extract is *Astragalus* polysaccharide [43]. A PDA medium prepared with *Roxanthus* pear juice was conducive to the growth of *P*. *ostreatus* mycelia and significantly increased the content of crude polysaccharides and total amino acids in the mycelia [44]. In the present study, the main carbohydrates of *P. tuber-regium* and *W. hoelen* sclerotium powder added were glucan and other polysaccharides [27,45]. By adding sclerotium powder to the PDA medium, the content of glucan and other polysaccharides was increased, and the carbon source in the medium was enriched, which may be highly suitable for the mycelial growth of *L. edodes* and *P. eryngii*.

Transcriptome data analysis of the PTR medium showed that the common PTR-induced differential genes of *L. edodes* and *P. eryngii* that were annotated in the GO database included cell response to chemical stimuli and enzyme activity. Metabolic pathways, such as starch and sucrose metabolism, glycolysis/gluconeogenesis, pantothenic acid, and CoA biosynthesis, were annotated in the KEGG database. These three metabolic pathways are closely related. After the carbohydrate metabolism of starch, sucrose, and glycolysis to form pyruvate, it combines with coenzyme A to produce a large amount of energy for growth and development. Pantothenic acid is a key precursor in the biosynthesis of coenzyme A, an important cofactor involved in various metabolic reactions, including the tricarboxylic acid cycle and fatty acid biosynthesis. The mechanism by which PTR promotes the growth of *L. edodes* and *P. eryngii* may be that the mycelium directly absorbs the components of the sclerotium powder for its own growth and development. The significant difference in gene expression induced by the PTR medium between the *L. edodes* and *P. eryngii* strains lies in the fact that *P. eryngii* was noted in the GO database for cell wall biogenesis, cell polysaccharide metabolism, and glucosidase activity, which verifies the hypothesis that *P. eryngii* can directly use the glucan from PTR. In terms of oxidoreductase activity, catalytic activity, haeme binding, and tetrapyrrole binding were noted in the GO database of *L. edodes* strain. The growth-promoting mechanism of PTR is speculated to promote the mycelia to utilise nutrients in the sclerotium by regulating activity.

Transcriptome data analysis in the WH medium indicated that *L. edodes* and *P. eryngii* showed common regulatory processes under WH-supplemented nutrient conditions. The common differential genes induced by WH in the two types of fungi were annotated in terms of the reaction to copper ions, copper ion binding, and oxidoreductase activity in the GO database, and in metabolic pathways such as starch, sucrose metabolism, and pyruvate metabolism in the KEGG database. Metal ions such as iron, copper, and manganese are essential for the maintenance of intracellular metal ion balance, act as cofactors in various enzymatic processes, and are an important part of the cellular metabolic machinery [46]. Multicopper oxidases constitute a regulatory system in which the oxidase activity maintains metal ion homeostasis [47]. The mechanism by which WH promotes the growth of *L. edodes* and *P. eryngii* might be through the increased activities of the multicopper oxidase family in the mycelia. The mycelia degraded organic matter in WH at a higher rate and increased the nutrient content in the medium, thereby accelerating mycelial growth. Among the regulatory pathways that differed between *L*. *edodes* and *P*. *eryngii*, the former was annotated in the KEGG database for alanine metabolism; cyano amino acid metabolism; alanine, aspartate, and glutamate metabolism; valine, leucine, and isoleucine degradation; and other metabolic pathways, which may indicate that the mushroom mycelium can also use the amino acid components in WH. *Pleurotus eryngii* was annotated in the KEGG for glycolysis, carbohydrate digestion, and absorption, further indicating that *P. eryngii* could utilise the polysaccharide components of WH.

Fungi possess the enzymatic mechanisms required to break down cell wall components [48,49,50] and are capable of producing related extracellular enzymes with hydrolysis and oxidation functions, such as CAZymes. Enzymes related to the carbohydrate degradation ability of lignoperoxidase, manganese peroxidase, and laccase are mainly found in lignocellulose, which can degrade lignocellulose into low-molecular-weight compounds that can be absorbed for fungal nutrition [51,52]. Carbohydrate family gene annotation of the differential genes of *L. edodes* and *P. eryngii* and cultured in the medium supplemented with sclerotium powder showed that the expression of the CAZyme family genes changed significantly. The expression level was mainly upregulated, and the enzyme activity was enhanced, which could effectively degrade carbohydrates in sclerotium powder, which may be one of the important mechanisms by which sclerotium powder promotes mycelial growth.

Combining the mycelial growth and transcriptome sequencing analyses, we found that although both edible mushrooms were able to utilise the carbohydrates in the sclerotia, the WH sclerotium had a greater impact on the mycelial growth than PTR, especially in the case of *L. edodes*. However, the content of polysaccharides in PTR is slightly lower than that of WH. It is unclear whether other components in the sclerotium, in combination with polysaccharides, are also influencing mycelial growth and how they exert this effect. Further research is needed to clarify this.

Our data indicated that adding sclerotial powder can significantly promote the mycelial growth of edible fungi. After adding a certain proportion of PTR and WH sclerotium powder, the mycelial growth rate increased, and the mycelia became denser, with the optimal addition ratio being 2%. It may be used to rejuvenate strains that have already undergone degeneration in the future. To the best of our knowledge, this study represents the first transcriptome profiling of the fungal sclerotia effect on the mycelial growth of edible mushrooms, providing new insights into the physiological mechanisms of substrate decomposition and nutrient metabolism in edible fungi. The transcriptome data indicated that different regulatory mechanisms were involved in the mycelial growth of *L. edodes* and *P. eryngii* depending on the sclerotial powder. It is possible that WH sclerotium powder may facilitate the degradation and uptake of nutrients by increasing the activity of the polycopper oxidase family. The addition of PTR sclerotium powder resulted in the enrichment of differentially expressed genes in starch and sucrose metabolism, glycolysis, and coenzyme A biosynthesis pathways in both mushrooms. The increased activity of carbohydrate-degrading enzymes in both mushrooms indicates that sclerotium powder may promote mycelial growth by efficiently degrading carbohydrates. However, the relationship between the differences in the promoting effects of different sclerotium powders on edible fungi and their constituent components is still unclear and needs further research.

## Data Availability

The raw reads obtained from Illumina NovaSeq in this study have been deposited into the NCBI Sequence Read Archive (SRA) database under accession number PRJNA1136801.

## Supplementary Materials

The following supporting information can be downloaded at: https://www.mdpi.com/article/10.3390/jof10120808/s1. Table S1: sequence comparison of proteins encoded by common DEGs; Table S2: primers used for the *Lentinula edodes* qRT-PCR; Table S3: primers used for the *Pleurotus eryngii* qRT-PCR.
