# Supplementary material for: Transcriptome Analysis of the Growth-Promoting Effect of Large Macrofungal Sclerotium Powder on Lentinula edodes and Pleurotus eryngii Strains
Source: J Fungi (Basel). 2024 Nov 21;10(12):808. doi: 10.3390/jof10120808 (PMC11679516; doi:10.3390/jof10120808)
Supplement: Supplementary file 1 [file jof-10-00808-s001.zip › jof-3240070-supplementary.pdf]

Table S1 Sequence comparison of proteins encoded by common differentially expressed genes.

Lentinula edodes		*Pleurotus eryngii*			
**Gene**	**Protein**	**Gene**	**Protein**	**score**	**E value**
GAW10075	Hypothetical protein	KAF9501075	Cytochrome P450	646	0
GAW10075	Hypothetical protein	KAF9486951	Cytochrome P450	349	$1.00e^{-98}$
GAW10075	Hypothetical protein	KAF9486888	Cytochrome P450	324	$3.00e^{-91}$
GAW09471	MFS general substrate transporter	KAF9497993	Hypothetical protein	202	$2.00e^{-54}$
GAW09471	MFS general substrate transporter	KAF9493880	Laccase	188	$3.00e^{-50}$
GAW09434	Cytochrome P450	KAF9501075	Cytochrome P450	186	$9.00e^{-50}$
GAW09434	Cytochrome P450	KAF9492298	Heme peroxidase	152	$2.00e^{-39}$
GAW09434	Cytochrome P450	KAF9499153	Hypothetical protein	143	$2.00e^{-37}$
GAW09299	Hypothetical protein	KAF9499881	Hypothetical protein	120	$8.00e^{-30}$
GAW09299	Hypothetical protein	KAF9499882	Copper transporter	117	$5.00e^{-29}$
GAW09299	Hypothetical protein	KAF9490413	Hypothetical protein	114	$1.00e^{-28}$
GAW09190	Transmembrane protein	KAF9497558	Hypothetical protein	110	$4.00e^{-27}$
GAW08854	Rta1-like protein	KAF9494720	peptide transporter MTD1	100	$8.00e^{-24}$
GAW08622	Glycoside hydrolase family 3 protein	KAF9500865	Hypothetical protein	95	$2.00e^{-22}$
GAW08213	MFS general substrate transporter	KAF9495632	Major facilitator superfamily multidrug-resistance	87	$5.00e^{-20}$
GAW08213	MFS general substrate transporter	KAF9496417	Oligopeptide transporter	86	$1.00e^{-19}$
GAW08213	MFS general substrate transporter	KAF9501075	Cytochrome P450	85	$2.00e^{-19}$
GAW08123	Hypothetical protein	KAF9499153	Hypothetical protein	83	$1.00e^{-18}$
GAW08123	Hypothetical protein	KAF9492795	Hypothetical protein	82	$2.00e^{-18}$
GAW08123	Hypothetical protein	KAF9487492	P-type ATPase-like protein	82	$4.00e^{-18}$
GAW07814	Poxa3b laccase small subunit	KAF9499519	Subtilisin-like protein	81	$8.00e^{-18}$
GAW07814	Poxa3b laccase small subunit	KAF9501268	Hypothetical protein	79	$2.00e^{-17}$

GAW07581	NAD -binding protein	KAF9492244	NAD(P)-binding protein	77	$8.00e^{-17}$
GAW07581	NAD -binding protein	KAF9499237	NAD(P)-binding protein	77	$1.00e^{-16}$
GAW07534	Glycoside hydrolase family 128 protein	KAF9501786	Hypothetical protein	62	$9.00e^{-13}$
GAW07534	Glycoside hydrolase family 128 protein	KAF9500865	Hypothetical protein	57	$2.00e^{-11}$
GAW07537	Family A1 protease	KAF9490640	Aspartic peptidase A1	58	$3.00e^{-11}$
GAW07537	Family A1 protease	KAF9498916	Acid protease	57	$8.00e^{-11}$

Table S2 Primers used for *Lentinula edodes* qRT-PCR.

Gene	Protein	Forward primer (5′→ 3′)	Reverse primer (5′→3′)
Actin	Actin	GCATCCTGTCCTTCTT ACCGAG	AAGAGCGAAACC CTCGTAGATG
GAV99068	Laccase lcc5	TGGTCGCACTTGTTC CGTCAG	TGGTATCGTCCGT CAACTCGTT
GAW00335	Manganese dependent peroxidase 1	TGCTGGACCTCACCT GCTACCT	TGGTGGGACTGGA GCAACAGAT
GAW03759	Laccase	CCTGCTGGTGCTGCG TTGAAT	CGGTGCGAGGTAG CTGACATTG
GAW05002	FAD-binding protein	GGGAGGAAATCGGT TCGGCATT	CGTGGCAAGGACA GCATTTGTG
GAW05592	Carbohydrate-binding module family 20 protein	GCTGCTGGCTGTGAT GCTCTT	CGTCCAGTGGCAA CACCTGAAT
GAW06442	Hypothetical protein	CGCATCAACACCAC CGATCCAT	TACGCCTGCCTCT GCCAATG
GAW07534	Endo-beta-1,3-glucanas e	ACAATGGCGACGGC GAGGTT	TTGGGCAACAGGG CTGGAGT
GAW08622	Glycoside hydrolase family 3 protein	GGTGAAGAATGCCG AGCCAAGG	GCTTGATCGTCGC ACTGACTCC

Table S3 Primers used for *Pleurotus eryngii* qRT-PCR.

Gene	Protein	Forward primer (5′→ 3′)	Reverse primer (5′→3′)
Actin	Actin	CCTTGGTCTTGAAGC TGCTG	GAGCGACGATCTTGA CCTTC
BDN71DRA FT_1435398	Hypothetical protein	GTGATGCTGCTTGCG GAGAAC	CGTCGTCTTCGGCGG TATAAAC
BDN71DRA FT_106407	Acetyl xylan esterase	CCACGACACCTCCAA CCACAAC	GGCGTTGAGAGCAG TGCATGT
BDN71DRA FT_1483600	Ferroxidase	GCCGCCTCCACCTAT TGATGTC	CTGTTACGCCAACCG CACCAT
BDN71DRA FT_1055494	Laccase	AGGTGTCCTTGTTCA CGGTTGG	TGACGAAGGCGGGT CCATCA
BDN71DRA FT_1447255	Carbohydrate-binding module family 13 protein	AGCAGTGGTTCCTCA CCGACAA	TCAACGGACCCAGG CTTGGTT
BDN71DRA FT_1445894	Carbohydrate-binding module family 13 protein	CGTTGACAGGTGTCC ACTTCGT	CGAATACCAAGCCG CCGATAGG
BDN71DRA FT_416606	Copper radical oxidase	GCTTCCTGCCACGGT CAACTT	CGAGTCGCTGGTCCA TGTGAAC
BDN71DRA FT_1607096	MnP-short, class II peroxidase	CGACTCCGTTGACAC CATCTTG	TGAGCAGCGACGGT ATGAGAG
